# IL-13 Promotes Collagen Accumulation in Crohn’s Disease Fibrosis by Down-Regulation of Fibroblast MMP Synthesis: A Role for Innate Lymphoid Cells?

**DOI:** 10.1371/journal.pone.0052332

**Published:** 2012-12-31

**Authors:** Jennifer R. Bailey, Paul W. Bland, John F. Tarlton, Iain Peters, Moganaden Moorghen, Paul A. Sylvester, Christopher S. J. Probert, Christine V. Whiting

**Affiliations:** 1 School of Veterinary Science, University of Bristol, Bristol, United Kingdom; 2 Mucosal Immunobiology and Vaccine Center (MIVAC), Department of Microbiology and Immunology, University of Gothenburg, Gothenburg, Sweden; 3 Molecular Testing, Innovation Centre, University of Exeter, Exeter, United Kingdom; 4 Histopathology Department, St Mark’s Hospital, Harrow, United Kingdom; 5 Department of Surgery, Bristol Royal Infirmary, Bristol, United Kingdom; 6 Department of Gastroenterology, University of Liverpool, Liverpool, United Kingdom; Universidade de Sao Paulo, Brazil

## Abstract

**Background:**

Fibrosis is a serious consequence of Crohn’s disease (CD), often necessitating surgical resection. We examined the hypothesis that IL-13 may promote collagen accumulation within the CD muscle microenvironment.

**Methods:**

Factors potentially modulating collagen deposition were examined in intestinal tissue samples from fibrotic (f) CD and compared with cancer control (C), ulcerative colitis (UC) and uninvolved (u) CD. Mechanisms attributable to IL-13 were analysed using cell lines derived from uninvolved muscle tissue and tissue explants.

**Results:**

In fCD muscle extracts, collagen synthesis was significantly increased compared to other groups, but MMP-2 was not co-ordinately increased. IL-13 transcripts were highest in fCD muscle compared to muscle from other groups. IL-13 receptor (R) α1 was expressed by intestinal muscle smooth muscle, nerve and KIR^+^ cells. Fibroblasts from intestinal muscle expressed Rα1, phosphorylated STAT6 in response to IL-13, and subsequently down-regulated MMP-2 and TNF-α-induced MMP-1 and MMP-9 synthesis. Cells with the phenotype KIR^+^CD45^+^CD56^+/−^CD3^−^ were significantly increased in fCD muscle compared to all other groups, expressed Rα1 and membrane IL-13, and transcribed high levels of IL-13. In explanted CD muscle, these cells did not phosphorylate STAT6 in response to exogenous IL-13.

**Conclusions:**

The data indicate that in fibrotic intestinal muscle of Crohn’s patients, the IL-13 pathway is stimulated, involving a novel population of infiltrating IL-13Rα1^+^, KIR^+^ innate lymphoid cells, producing IL-13 which inhibits fibroblast MMP synthesis. Consequently, matrix degradation is down-regulated and this leads to excessive collagen deposition.

## Introduction

Inflammation-induced fibrosis – pathologic accumulation of extracellular matrix (ECM) – in the intestine represents a serious complication of inflammatory bowel diseases (IBD). In ulcerative colitis (UC), ECM may accumulate in the mucosa and submucosa (SM) contributing to stiffening of the colon, whereas in Crohn’s disease (CD) the excess ECM, particularly fibrous collagen, deposited transmurally, leads to stricture and loss of normal function [1]. Up to one third of patients with CD develop debilitating intestinal fibrosis.

Inflammation in CD may lead to fibrosis by up-regulation of pro-fibrogenic factors, such as TGF-β [2]. Fibroses in lung, liver and kidney have been linked to T cell synthesis of IL-13 [3]. This can promote collagen synthesis independently of TGF-β [4]; and cause smooth muscle cell proliferation [5], [6] and contraction [7], which may contribute to fibrotic stricture formation. IL-13 activates many other cells, including macrophages, mast cells, B cells and nerve cells (reviewed [8]), potentially contributing to pathology.

IL-13 signals predominantly via the low affinity IL-13Rα1 which forms dimers with IL-4Rα and subsequently activates the JAK1/STAT6 pathway. IL-13Rα2, originally considered to act as a decoy receptor, has recently been shown capable of signalling [9] and can occur in cell surface and soluble forms. TNF-α, in conjunction with IL-13, was shown to increase IL-13Rα2 synthesis in macrophages leading to TGF-β synthesis [9]. In a mouse model of intestinal fibrosis, blockade of IL-13Rα2 and TGF-β signalling reduced levels of colonic IGF-I and collagen deposition [10]. IL-13 promotes transcription of matrix metalloproteinase (MMP)-2, 9, 12 and 14 [11]; decreases MMP-1 synthesis [12]; and synergises with TGF-β to increase fibroblast tissue inhibitor of metalloproteinase (TIMP)-1 [13] – a pro-fibrotic mechanism. IL-13 has recently been linked to fistula formation in CD [14]. Consequently, both IL-13 and IL-13Rα2 are considered potential therapeutic targets in fibrotic diseases and in other CD pathologies [9], [14].

Fibrosis occurs when extracellular matrix (ECM) synthesis exceeds degradation. Breakdown of collagen, the principle ECM molecule deposited in fibrosis, is mediated by proteolytic enzymes. Of these, MMPs are principally involved: collagenase (MMP-1), cleaves mature collagen fibres [15]: and MMP-2 is co-ordinately regulated with collagen synthesis via TGF-β but also through common promoter elements such as AP-2 and SP1, and probably serves to remodel nascent collagen molecules to allow correct fibril formation [16], [17], [18]. Other MMPs may play a role in inflammation-induced fibrosis, for example MMP-9, which mediates leukocyte migration, and MMP-14, which may promote fibrosis via up-regulated TGF-β signalling [19]. MMP activity is controlled by specific inhibitors, the TIMPs, levels of which are modulated in disease processes. Therefore it is important to attempt to understand the complex interplay between these mediators which determine the level of collagen deposition.

Much of the work identifying IL-13 as a fibrotic mediator has been carried out in mouse models and in tissues other than the intestine. In order to identify the IL-13 pathway as a relevant therapeutic target in CD, it is important to understand the processes occurring in human intestine. We have therefore investigated the hypothesis that CD fibrosis results from an IL-13-mediated imbalance in collagen synthesis and degradation. The results indicate that IL-13 is elevated in fibrosis, resulting in decreased MMP-2, MMP-9 and MMP-1 activity which would promote collagen deposition.

## Methods

### Ethics Statement

Samples were collected from Bristol Royal Infirmary and written informed consent was obtained by all patients. The study was carried out under Ethical Approvals E4896 (Central and South Bristol Ethics Committee) and 07/H0205/44 (Somerset Research Ethics Committee).

### Reagents

All chemicals and reagents were obtained from Sigma (Poole, UK) unless stated otherwise.

### Patients and Samples

Intestinal tissue was collected from CD patients undergoing surgery for bowel obstruction (n = 19, 12 men and 7 women, age range 18–73 y, average age 35 y), including uninvolved areas; ulcerative colitis (n = 8, 6 men and 2 women, age range 18–62 y, average age 32 y), including uninvolved areas; or colorectal cancer (n = 13, 7 men and 8 women, age range 56–88 y, average age 72 y), described in Table S1. Tissues were examined macroscopically and palpated to determine areas of fibrosis, and subsequently examined by microscopy and allocated to “uninvolved” and “fibrotic” groups. Tissue evaluations were carried out independently by two “blinded” observers (MM and PB). From each sample, full-thickness tissue was frozen for immunohistology. As the excessive collagen deposition occurs in the submucosa and outer muscle layers, tissue was separated into muscle-enriched (referred to as “muscle”) and mucosa-enriched (“mucosa”) fractions. The muscle was pinned and the mucosa dissected out below the muscularis mucosa. In some fibrotic samples, the separation was carried out within the submucosa approximately 1–1.5 mm from the luminal surface, carefully excluding contamination of muscle by mucosa. Fractions were snap frozen in liquid nitrogen and stored at −80°C; or in RNA later, pending biochemical analyses. A sample of each fraction was orientated in OCT before freezing in liquid nitrogen. Image and qPCR analysis were carried out on a subset of patients (n = 8 for each group for cancer, inflamed UC, uninvolved CD and fibrotic CD; n = 3 for uninvolved UC).

### Sample Preparation

Frozen tissue fractions were pulverised in a liquid nitrogen-cooled freezer mill and homogenised in immuno-precipitation (IP) lysis buffer (300 mM NaCl, 0.05 M Tris pH 7.4, 1% Triton X-100) or in RIPA buffer (1% NP40, 1% sodium deoxycholate, 0.1% SDS, 0.15 M NaCl, 2 mM EDTA, 0.01 M phosphate buffer pH 7.2) for 1 h on ice, with 10 strokes of the homogeniser. Extraction buffer contained protease inhibitor cocktail (P8340) and phosphatase inhibitors cocktails 1 (P2850) and 2 (P5726).

### Cell Culture

Primary intestinal fibroblasts, isolated from uninvolved intestinal muscle tissue taken from CD and UC patients, were cultured as previously described [20], with some modifications. Briefly, 2×2 mm separated muscle were incubated in 100 u/ml type IV collagenase for 2–3 h and fibroblasts allowed to adhere overnight in DMEM containing 10% FCS, non-essential amino acids, vitamins, L-glutamine, nystatin and double strength antibiotics (penicillin 200 u/ml, streptomycin 200 µg/ml, amphotericin 0.25 µg/ml, gentamicin 100 µg/ml). Fibroblasts were transferred to 1× antibiotics after 7 days. This medium was then used for all experiments and cell culture, except when cells were starved and cytokine-treated, when the medium contained 0.1% FCS and 250 µg/ml ascorbate-2-phosphate, essential for collagen synthesis. Cells (2500/well of a 96-well plate) were cultured for 48 h in 10% FCS, before starvation for 48 h, and then incubated in TGF-β (2 ng/ml, AbDSerotec, Kidlington, UK), TNF-α (10 ng/ml, AbDSerotec), or IL-13 (20 ng/ml, AbDSerotec) for 72 h. Collagen, TGF-β and MMPs were assayed from frozen supernatants. For each line and each experiment, a titration of cell number was performed and standard curve prepared on cells grown for 16 h and assayed by MTT (3-(4,5-dimethylthiazol-2-yl)-2,5-diphenyl tetrazolium bromide) (Millipore, Watford, UK). For signalling analyses, cells were added to 12-well plates, cultured until 80% confluent, starved for 48 h and then cytokines added in starvation media for 0.5 to 72 h.

### Explant Culture

Muscle fragments (2×2 mm), prepared as above, were washed in serum-free medium and 3–5 fragments per well were placed in 12-wellplates and covered with 1 ml of starvation medium, with or without 20 ng/ml IL-13. Explants were cultured for up to 24 h and then snap frozen in liquid nitrogen with or without OCT.

### ELISA

Pro-MMP-1 was measured in culture supernatants by ELISA (R&D Systems, Abingdon, UK); TGF-β1 was measured in culture supernatants by ELISA (in-house) [21]; and collagen synthesis was measured in culture supernatants by ELISA for type I collagen C-terminal pro-peptide (CICP) (Technoclone, Dorking, UK). Assays were run as 5 well replicates. Tissue extracts were analysed in duplicate for CICP and MMP-1 using the same assays as culture supernatants, and IL-1β and TIMP-1 were analysed using R&D Systems ELISA kits. Data were normalized to soluble protein for tissue extracts or to cell number for culture supernatants. Levels were determined using standards and data expressed as the ratio to unstimulated cultures.

### Western Blotting and Zymography

Adherent cells were washed in cold TBS and lysed in RIPA buffer (40 µl/well). Explants were weighed and homogenised in RIPA buffer (500 µl:100 mg wet weight). Lysates or tissue extracts were centrifuged and supernatants containing equal amounts of soluble protein (cells 0.01 mg/lane; explants 0.05 mg/lane; original extract for IP samples 3 mg/lane) were subjected to SDS-PAGE (8%) and proteins transferred to Immobilon P. Blots were blocked and proteins detected using rabbit antibodies to STAT6 (1∶1000), or PSTAT6 (1∶1000) (Table S2) and donkey anti-rabbit peroxidase (Jackson, 1∶20000) by chemiluminescence (ECL plus, GE Healthcare, Little Chalfont, Bucks, UK) and Biomax light film. MMP-2 and MMP-9 were assayed by gelatin-zymography as described previously [22]. Bands were quantified using NIH image.

### RNA Isolation

Muscle fragments were disrupted using a TissueLyser (Qiagen Ltd, Crawley, UK) followed by treatment with proteinase K (6 mAU/ml, 55°C/15 mins) and RNA extracted using Macherey-Nagel NucleoSpin RNA II Isolation Kits (ABgene, Epsom, UK). The resulting RNA was assessed for quantity and quality by automated gel electrophoresis (Experion, Bio-Rad Laboratories, Hemel Hempstead, UK) and stored at −80°C before use.

### Primer and Hydrolysis Probe Design

Primers and probes were designed using Primer 3 [23] (http://frodo.wi.mit.edu/primer3) and M-Fold using the human specific GenBank sequences for IL-13, IL-13Rα2, type I collagen and TGF-β1 primer sequences, GenBank accession numbers and housekeeper genes (primers as [24]) are given in Table S3a and Table S3b. Primers and probes were synthesised by Metabion International AG (Munich, Germany).

### Quantitative RT-PCR (qPCR)

Duplicate RNA samples, confirmed as free from genomic contamination, were subject to reverse transcription using random hexamers and ImProm-II Reverse Transcription System (Promega Corporation, Southampton, UK). Quantitative PCR was performed using HotStarTaq Master Mix (Qiagen). Gene specific amplification was performed using 4.5 mM MgCl_2_, 0.2 µM primer, 0.1 µM probe or SYBR Green 1 (1/100,000) and cDNA equivalent to 35 ng RNA. Sample incubations were performed in an MxPro3005P (Stratagene, California, USA) at 95°C for 15 minutes and then 45 cycles of 95°C for 15 seconds, and 60°C for 30 seconds. When SYBR Green I was used, samples were heated from 75°C to 95°C in 0.5°C increments with a dwell time at each temperature of 10 seconds. Threshold cycle (Ct) values were calculated when the sample exceeded baseline fluorescence mean ±10 SD. A negative control of nuclease-free water and a positive control sample with a known Ct value were included with all sample runs.

In order to determine the most appropriate housekeeper genes for the study, all 8 potential genes were quantified in all samples. Relative copy number was calculated using the E^ΔCt^ method (E: reaction efficiency) with ΔCt values calculated from the sample with the largest Ct (fewest gene copies). The geNorm VBA applet for Microsoft Excel was used to determine the most stable genes. The four most stable genes for muscle fractions were (in order of stability) RPL13A, YWHAZ, SDHA and GAPDH. These genes were then used for the analyses.

Relative expression data for each of the gene targets was calculated using the qBase applet for Microsoft Excel (http://medgen.ugent.be/qbase/). This applet calculates a relative copy number for each sample, normalised against the four stably expressed housekeeper genes, using the methods described by Vandesompele *et al*. [24]. All samples were normalised to the sample with the latest Ct value (assigned a relative copy number  = 1).

### Histology and Immunofluorescence

Formalin fixed tissues were stained with Haematoxylin van Giesons (HVG) stain for collagen and examined for the presence of mucosal inflammation and the presence of fibrosis.

Acetone-fixed frozen sections (5 µm) of full thickness tissue or cytokine-treated explants were blocked in 10% normal goat and/or donkey serum followed by avidin/biotin blocking (Vector Laboratories, Peterborough, UK). Sections were incubated overnight at 4°C with primary antibodies (Table S2). The isotype-specific secondary antibodies to mouse IgG (all used at 1∶100 from Southern Biotechnology) were all raised in goat and were conjugated to biotin, Texas red, FITC, AF633, or TRITC, and combined according to primary antibodies applied. Donkey anti-rabbit IgG (1∶400), streptavidin-Texas red (1∶100) and streptavidin-Dylight488 (1∶200) were obtained from Stratech (Newmarket, Suffolk, UK). Species and isotype-specific negative controls were used for each experiment. Finally, sections were mounted using Vectashield (Vector Laboratories), viewed using a Leica fluorescence microscope and digital images captured using Image Pro Plus software (Media Cybernetics, Silver Spring, MD).

### Laser Capture Microscopy

Eight-micron frozen sections were collected onto polyethylene naphthalate (PEN)-coated slides (Leica Microsystems). A modified staining procedure was used to maximize RNA recovery from captured cells: sections were fixed in ice-cold ethanol; primary mouse anti-KIR and secondary goat anti-mouse FITC were combined in a buffer containing RNALater and applied to sections for 2 h on ice; after a brief wash in PBS containing RNALater, sections were dehydrated in ethanol, viewed without a coverslip and held on dry ice before LCM. KIR^+^ cells were identified by fluorescence imaging in the Zeiss PALM Microbeam, cut using minimal laser beam diameter and minimal profile diameter and catapulted into adhesive caps of microtubes. 500 KIR^+^ cells and 500 KIR^−^ adjacent tissue fragments were captured in each experiment and immediately processed for RNA extraction using a Qiagen RNeasy Micro RNA extraction kit. cDNA was generated and amplified by Whole Genome Amplification (Qiagen), followed by RT-qPCR using the Roche Light Cycler 480 with primers as detailed above. Fold differences in IL-13 transcription were assessed against the GAPDH housekeeper. Due to the low levels of RNA extracted using this technique it was only possible to normalize against one housekeeper gene, GAPDH, one of the most stably expressed genes.

### Image Analysis

Images, 5–10 per section, were captured at ×20 and analysed using ImageJ software as previously described [25]. Amount of contaminating submucosa attached to tissue fractions was assessed by analysing the area of submucosa as a percentage of the whole fragment area in the subset of tissues described in “patients and samples”.

### Statistics

The data were tested for normality using P-P plots and then analysed by bivariate analysis and Pearson correlation on samples paired by patient, or ANOVA on untransformed or log transformed data for all five tissue groups, followed by post-hoc analysis. Cell culture data were analysed by ANOVA and post-hoc analysis. Data were considered significant below p = 0.05.

## Results

### Strictures in Crohn’s Disease Involve Increased Collagen Deposition in Gut Layers External to Mucosa

**Figure 1:**
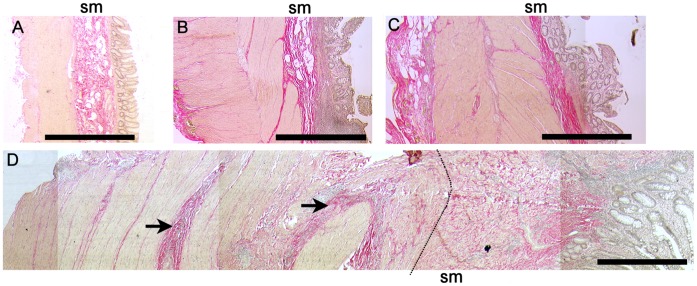
Increased collagen synthesis in muscle and submucosa in fibrotic CD tissue. Full thickness sections of formalin fixed tissue stained with Haematoxylin van Geisens. Collagen is stained bright pink and nuclei are grey. (A) Cancer control (colon), (B) Inflamed UC (C) Uninvolved Crohn’s disease (terminal ileum) and (D) Crohn’s disease with stricture (terminal ileum) (stitched image). Arrows show dense collagen deposits in outer muscle layers. Dotted line defines the outer muscle/sub-mucosa (sm) border. Images captured with ×2.5 objective. Bars represent 1 mm.

In histologically normal cancer controls, uninvolved CD or UC tissues, collagen was distributed in thin dense bands between muscle bundles, throughout the loose connective tissue of the submucosa, and in the serosa (Figure 1). A similar pattern was observed in inflamed UC. Fibrotic CD intestine, from both small and large bowel, was defined as a sample from a CD patient receiving surgery for a diagnosed stricture which, on histopathological assessment, had increased and/or disrupted collagen deposition, with or without obvious muscle hyperplasia. Most (13/19) fibrotic CD samples had evidence of inflammation in the mucosa and/or muscle. In fibrotic CD, collagen deposition was variable; the submucosa and/or serosa were often expanded, with dense collagen between the muscle bundles. In some strictures, the intestinal wall was considerably thickened. Dense collagen deposition was not observed within the mucosa.

To examine changes in factors within the intestine where excessive collagen is deposited, namely the gut layers external to the mucosa, without confounding results with elements derived from the leukocyte and epithelial-rich mucosa, we separated mucosa away from muscle layers. After separation, there was no contamination of muscle samples by mucosa, as judged by microscopy (Figure S1). The boundary between the submucosa and muscle layer was less distinct in fCD than in other groups, so this was defined as the line where discernable muscle bundles ended. The submucosa remained attached in variable amounts to muscle and mucosa fractions and, by image analysis, there was a relatively larger amount of contamination of the mucosa fraction (Figure S1A,B). There was no significant difference between the percentage of submucosa attached to the muscle when fCD was compared to cancer (p = 0.06), uUC (p = 0.08), uCD (p = 0.05) or iUC (p = 0.05). When muscle data from all groups were combined, the amount of attached submucosa significantly correlated with collagen synthesis, pro-MMP-1 and TIMP-1 (p<0.04 all comparisons) but not with IL-1β, pro-MMP-2 proteins or IL-13 mRNA. There were no correlations between any of these factors and mucosa-attached submucosa.

### In Fibrotic Muscle, Collagen Synthesis is Increased without a Corresponding Increase in the Remodelling Enzyme, MMP-2

**Figure 2:**
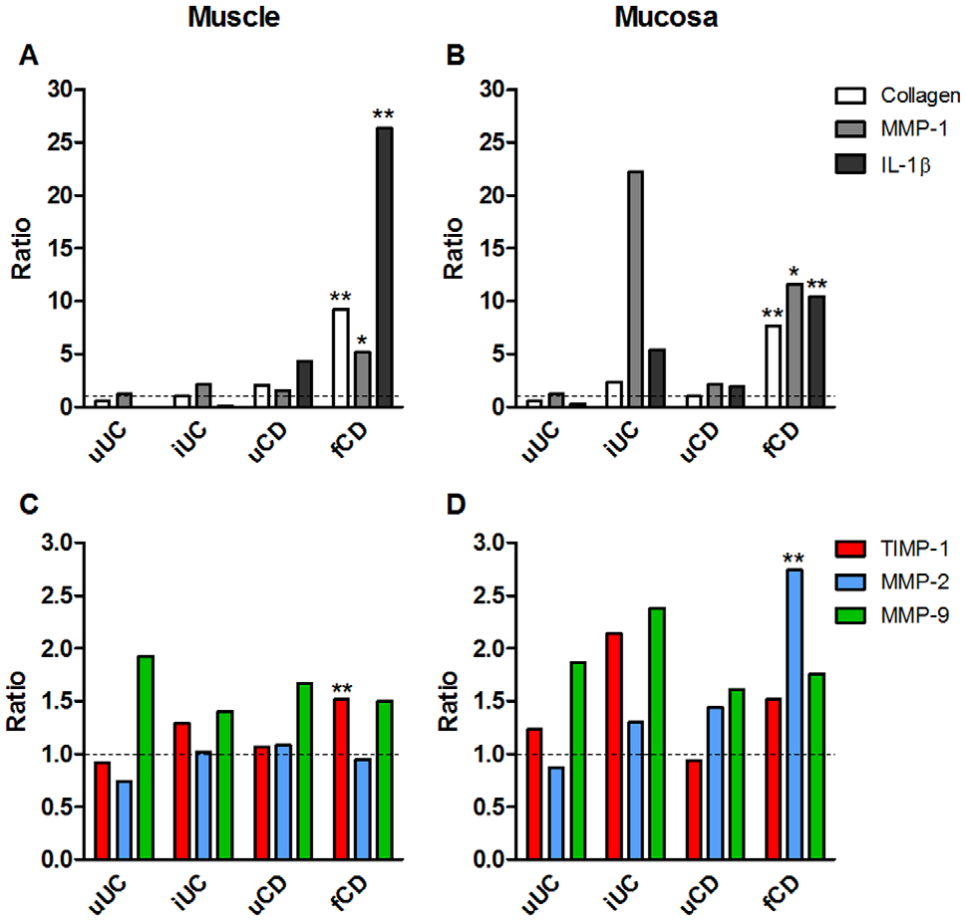
Fibrogenesis is increased in CD muscle and mucosa tissues, without concomitant MMP-2 synthesis in muscle. Tissue fragments were extracted and analysed by ELISA (collagen CICP synthesis, IL-1β, MMP-1, TIMP-1, TIMP-2) or zymography (MMP-2, MMP-9). Data are expressed as the ratio of the group mean for uUC, iUC, uCD or fCD to the mean for cancer control tissue for each parameter. Data are derived from 14–15 cancer controls, 3 uUC, 8 iUC, 8–12 uCD and 14–21 fCD patients. See Table S4 for actual means and SEM for each parameter. *p<0.05, **p<0.01 to cancer controls.

Type I collagen synthesis was significantly greater in fibrotic CD muscle compared to all other samples (p<0.01 all comparisons) (Figure 2A,B, Tables S4 and S5a). ProMMP-2 was not increased in fibrotic CD muscle compared to other groups (p>0.33 all comparisons) and the levels of proMMP-2 did not correlate with collagen synthesis in the muscle (p = 0.72 for all groups, p = 0.39 fCD only) (Table S5b). ProMMP-1 was significantly increased (p = 0.04) compared to cancer control tissue and TIMP-1 was significantly higher than all other groups (p<0.04 all comparisons) in fibrotic CD muscle. When fCD was compared to uCD, there was a highly significant increase in TIMP-1, collagen synthesis and IL-1β in fCD extracts (<0.001 for all comparisons). MMP-9 was elevated compared to cancer control tissue in all groups, but this was not significant in any comparison.

By comparison, in mucosa from fibrotic CD (Figure 2 C,D), there was a significant increase in collagen synthesis and MMP-2 compared to cancer controls (p<0.02) and compared to uCD (p<0.01), and pro-MMP-2 levels did correlate with collagen synthesis (p = 0.003) (Table S5b). There was also a significant increase in TIMP-1 and pro-MMP-1, compared to uCD. In mucosal tissue from inflamed UC (Figure 2C,D), collagen synthesis, pro-MMP-1, TIMP-1, pro-MMP-9 and IL-1β were significantly increased (p<0.03, all comparisons) compared to cancer control tissues. The increased level of mucosal MMP-1 in iUC tissues compared to fCD tissue approached significance (p = 0.056).

Figure 2 shows the ratio of the various parameters to cancer control tissue, Table S4 shows the means and SEM of the data, Table S5a shows the trends and levels of significance and Table S5b shows the correlations between collagen synthesis and various parameters.

### Raised IL-13 and IL-13Rα2 Transcription in fCD Muscle

**Figure 3:**
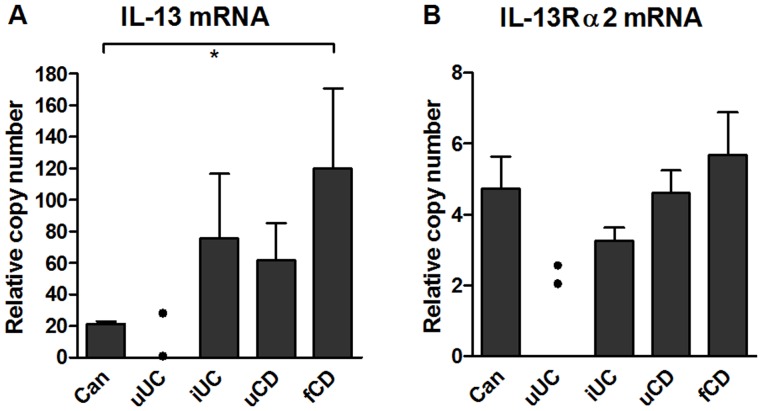
IL-13 transcription, but not IL-13Rα2 transcription, is increased fibrotic muscle. (A) IL-13 mRNA and (B) IL-13 Rα2 mRNA in muscle tissue lysates. RNA was extracted from tissue fragments and processed for qPCR. Results were normalized to four stably expressed housekeeper genes. Data are derived from 14–15 cancer controls, 2 uUC, 8 iUC, 8–12 uCD and 14–21 fCD patients. Significant differences from fibrotic CD, *p<0.05.

In tissue extracts by qPCR, IL-13 mRNA (Figure 3A) was increased in fibrotic CD muscle, and this was significant compared to cancer (p<0.05). There was a trend towards increased IL-13Rα2 transcription in fibrotic CD muscle compared to inflamed UC (p = 0.055) but not to other groups (Figure 3B). It was not possible to quantify IL-13 or receptors in any tissue extracts because of endogenous inhibitory factors, and IL-13 Rα1 transcription was not determined.

### Phenotype of IL-13Rα1 and IL-13Rα2^−^ Expressing Cells

**Figure 4:**
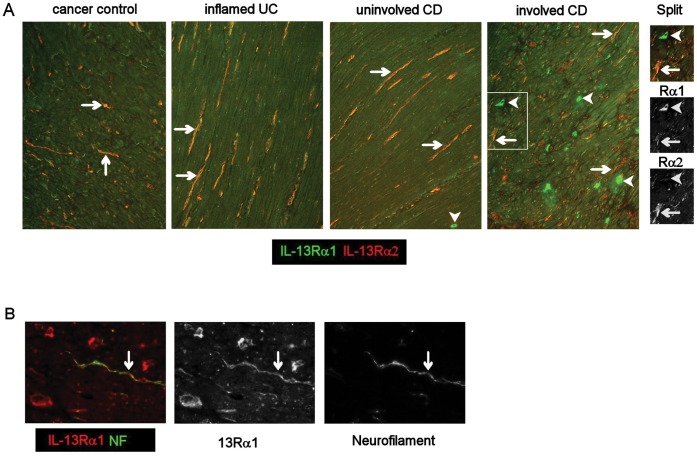
IL-13 receptors are expressed on smooth muscle cells and neurofilaments in CD strictures. Frozen sections were processed for double immunoflorescence. (Panel A) double labeling for IL-13 Rα1 and Rα2 in intestinal muscle tissue, arrows show double-stained cells, arrowheads show cells expressing Rα1 only at a very high level. Boxed area is shown as the colour image and its monochrome split. (B) Co-staining of IL-13Rα1 and neurofilament (NF) (green) and the monochrome split images. A,B, ×20 images.

**Figure 5:**
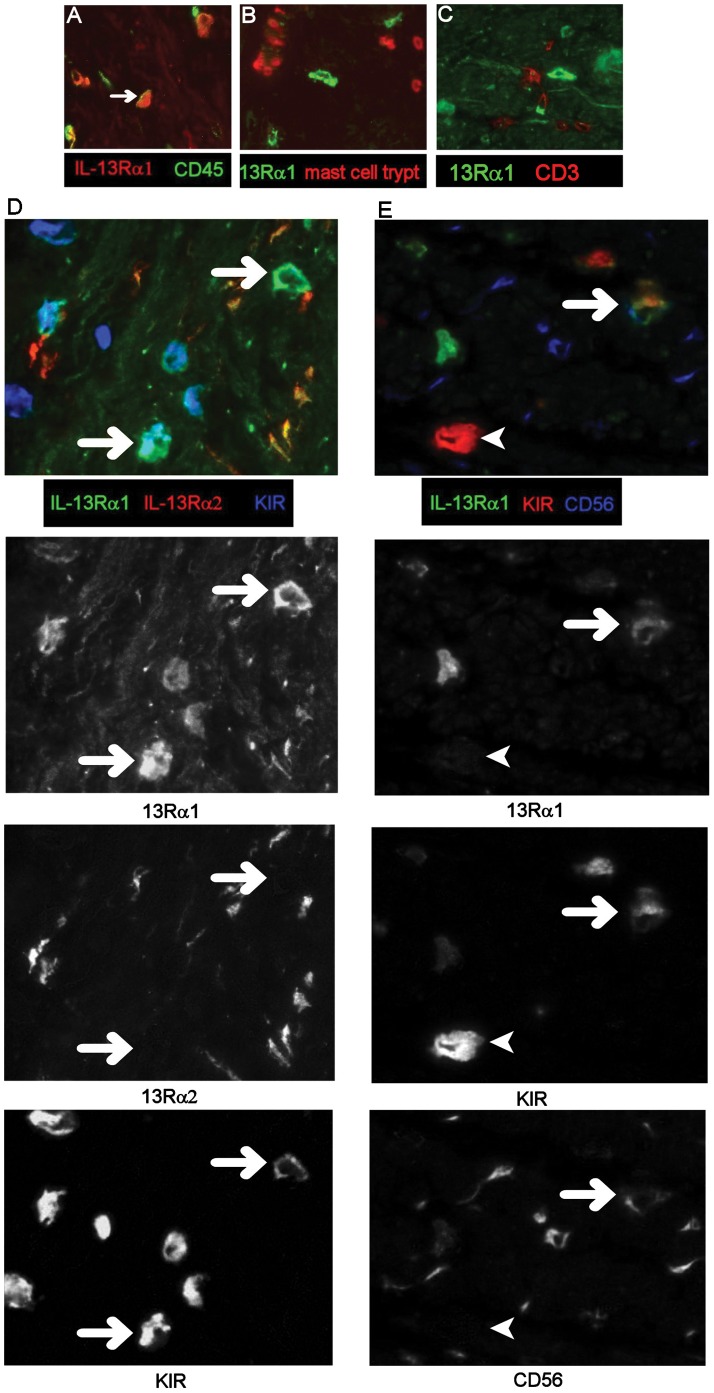
Mononuclear cells expressing very high levels of IL-13Rα1 co-express CD45, KIR and CD56. Images of frozen, immunostained tissue were captured using a 12 bit monochrome camera and images from the different colour channels combined to form two or three colour images. Multicoloured composites D and E, have been split into their original monochrome images to aid interpretation. Arrows show Rα1 co-localising with other markers. 13Rα1 co-localises with CD45 (A) but not mast cell tryptase (B) or CD3 (C). Panel D, 13Rα1 (green), 13Rα2 (red) and KIR (blue), plus split images for 13Rα1, 13Rα2 and KIR, note that 13Rα2 does not colocalise with 13Rα1^+^KIR^+^ cells (arrows). Panel E, 13Rα1 (green), KIR (red) and CD56 (blue). Split image shows a cell expressing all three markers (arrow), note there is also a strongly KIR-expressing cell (bottom left of image) which does not express 13Rα1 or CD56 A–C, ×40 images, D and E, ×64 images. Representative images from 8 patient samples.

**Figure 6:**
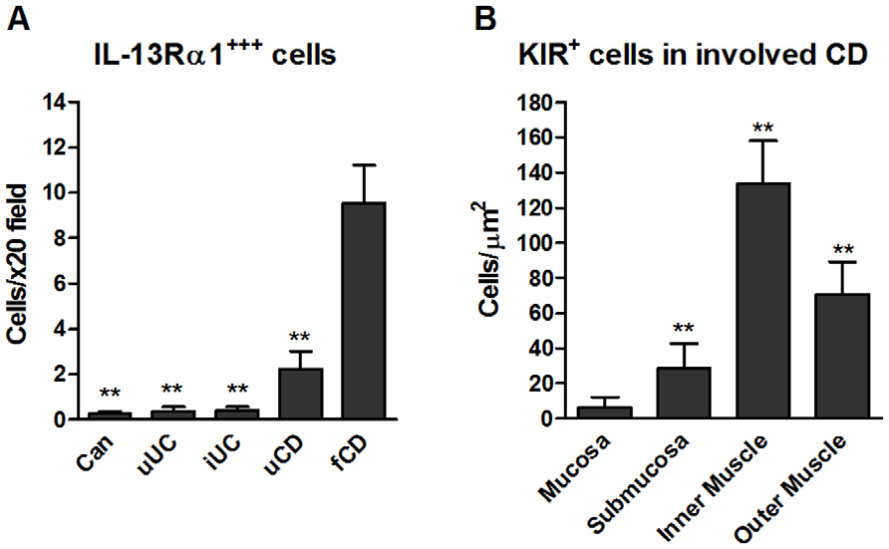
IL-13Rα1 and KIR expressing-cells are increased in fibrotic CD, particularly in the muscle, by image analysis of immunostained frozen tissue sections. (A) Total number of mononuclear cells/field expressing very high levels of Rα1 and no co-expression of Rα2 in muscle tissue (**p<0.01 to fCD), n = 8 for all groups except n = 3 for uUC. (B) Distribution of cells expressing very high levels of KIR in involved CD tissue. Data are the mean+SD of from 3 patients (20 images/patient), (*p<0.01 all comparisons).

**Figure 7:**
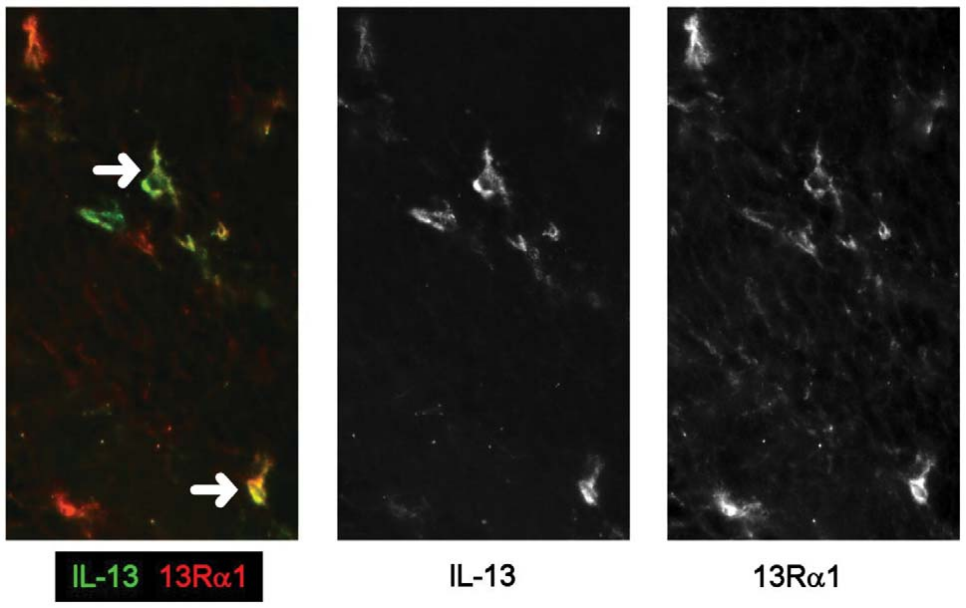
IL-13 is co-localises with IL-13Rα1 in fCD muscle. Double labeling, in frozen fibrotic CD tissue sections, for IL-13 (green) and IL-13Rα1 (red) and split monochrome images (×20).

In all groups, IL-13Rα1 and Rα2 were co-expressed (Figure 4A) by neurofilament^+^ (Figure 4B) spindle-shaped cells in the muscle layers and IL-13Rα1 was expressed at low levels on smooth muscle cells (Figure 4A). IL-13Rα1 was highly expressed on a population of mononuclear cells in the muscle in CD (Figure 4A). These Rα1^+^ cells expressed variable levels of CD45 (Figure 5A) but did not express mast cell tryptase (Figure 5B), CD3 (Figure 5C), mast cell chymase (data not shown) or NKG2D (data not shown). Anti-NKp46 antibody stained smooth muscle cells in all groups even at high dilution, such that no evidence of co-localisation with Rα1 was obtained. Most of these Rα1^+^ cells co-expressed KIR (Figure 5 panel D and E), and a proportion expressed CD56/NCAM (nerve cell adhesion molecule) (Figure 5 panel E) to a variable extent. The majority of cells expressing KIR also expressed IL-13Rα1 (KIR^+^ Rα1^+^ cells, mean 77% ±23%SD; KIR only, mean 10% ±10%SD; Rα1 only mean 13% ±9%SD, of cells/x20 field (data from 4 patients and 3 fields per tissue)). These IL-13Rα1^+^ cells were highly significantly increased (p<0.001) in muscle from fibrotic CD intestine compared to all other groups (Figure 6A) and were rarely observed in muscle tissue from cancer control or inflamed UC intestine (Figure 4 panel A, Figure 6A) and there was an uneven distribution of KIR^+^ cells in fibrotic muscle tissue, with the highest numbers in the inner muscle, and significantly fewer in the mucosa compared to all other regions (p<0.01 all comparisons) (Figure 6B). Variable levels of IL-13 co-localised with the IL-13Rα1^++^ cells (Figure 7) and there was no evidence IL-13 expression by other cells in the muscle or submucosa. It was not possible to analyse whether IL-13 co-localised with KIR as the primary antibodies were both IgG1. In all experiments, isotype matched control antibodies were used, and no staining was observed in muscle tissue.

By image analysis, the highest levels of IL-13Rα1 were observed in fibrotic CD muscle (Figure S2A) and total IL-13Rα2 was significantly increased (p<0.01) in fibrotic CD muscle when compared to all other groups (Figure S2B). The highest double positive (IL-13Rα1^+^Rα2^+^) area was observed in the muscle layers of fibrotic CD (Figure S2C).

### KIR^+^ Cells Synthesis IL-13

While the majority of KIR^+^ cells expressed high levels of IL-13Rα1, and IL-13Rα1^+^ cells expressed cell surface IL-13, it was not known whether KIR^+^ cells also produced IL-13. Therefore, a protocol was developed for the isolation, by laser capture microscopy (LCM), of KIR^+^ and adjacent KIR^−^ cells from fibrotic muscle tissue. Preliminary to LCM analysis, comparison was made between transcription of IL-13 in fibrotic CD tissue in two patients, and non-fibrotic CD tissue or uninvolved UC tissue, using whole tissue section extracts, as used in the LCM protocol (Table 1). IL-13 transcripts were readily detected in tissue from CD fibrotic gut, but were at a significantly lower level in tissue from non-fibrotic CD or UC gut (Figure S3). Levels of IL-13 transcript were consistently greater in KIR^+^ cells compared to adjacent KIR^−^ cells from each of these fCD muscle samples but both samples had similar GAPDH transcript levels (Table 1, Figure S3). Overall, IL-13 transcript levels were 114.8+/−3.4 times greater in KIR^+^ cells than in KIR^−^ cells. Interferon-γ from NK cells has been shown to be a regulator of liver fibrosis [26]. However, analysis of frozen tissue sections of fibrotic intestine from these two patients, both of which had high level muscle infiltration by KIR^+^ cells, showed that IL-13 transcription was readily detectable (Table 1, Figure S3), whereas IFN-γ was undetectable in both samples. In summary, the KIR^+^ cells we have described within CD fibrotic muscle are a major source of IL-13, but do not transcribe IFN-γ.

**Table 1:** KIR^+^ cells in fibrotic muscle tissue synthesise IL-13.

	Whole tissue^1^			KIR^+^ cells^2^		KIR^−^ cells^2^	
Sample	IL-13	GAPDH	IFN-γ	IL-13	GAPDH	IL-13	GAPDH
fCD1	32.50	29.98	∞	35.03	31.62	41.78	30.95
fCD2	36.85	30.03	∞	35.97	30.84	42.90	30.43
iUC1	41.52	29.99	ND	ND	ND	ND	ND
uCD1	40.45	31.26	ND	ND	ND	ND	ND

Data are Ct values from a representative set of experiments.

qRT PCR on whole tissue section extracts^1^, or qRT PCR on RNA derived from laser captured KIR^+^ or KIR^−^ cells^2.^

fCD, fibrotic CD; iUC, inflamed UC; uCD, uninvolved CD; ND not done.

### Explanted Muscle Tissue Responds to IL-13

**Figure 8:**
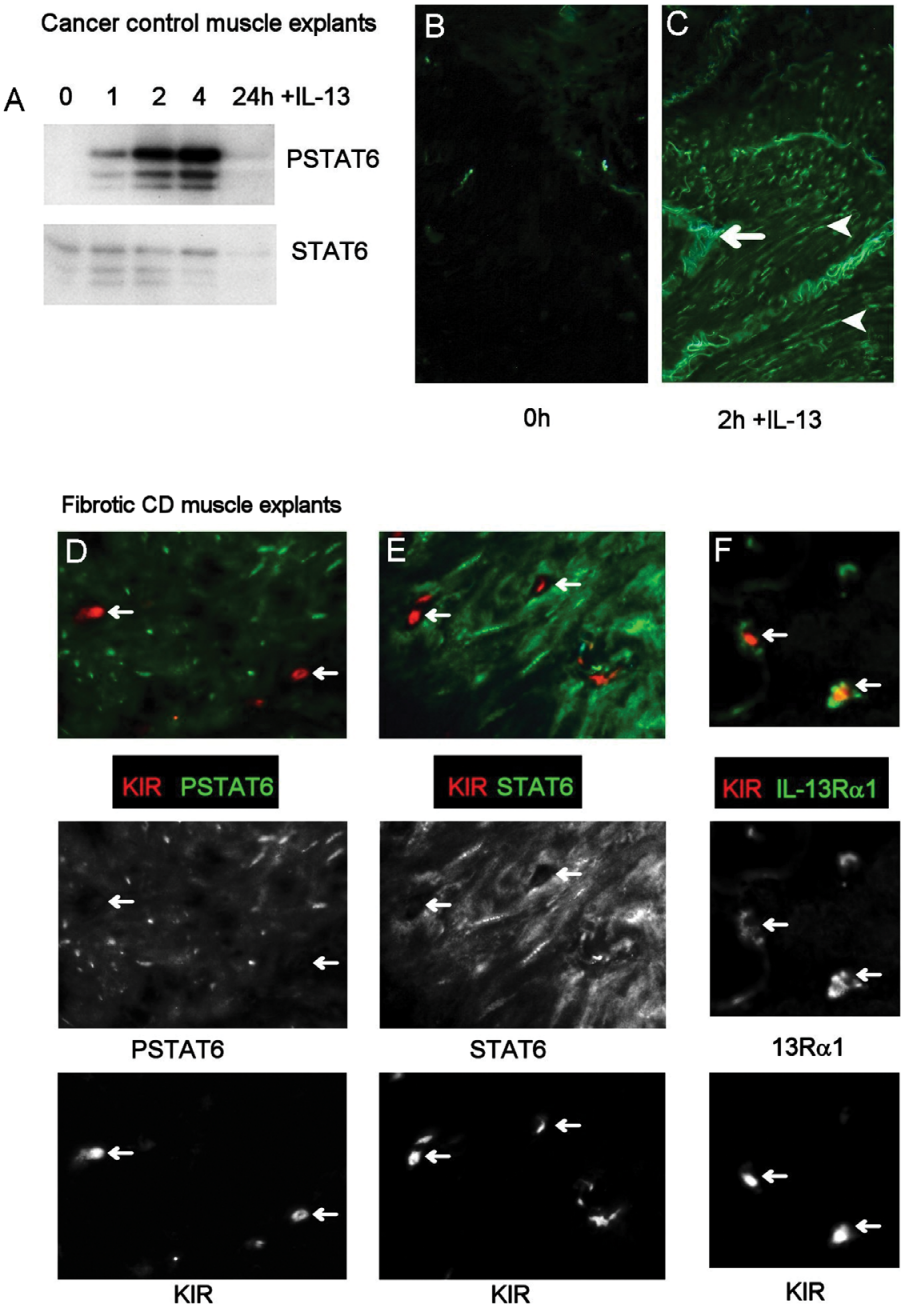
IL-13 activates smooth muscle cells, but not KIR^+^ cells, in tissue explants. Tissue explants were cultured with or without IL-13 for 1–24 h. Explants were then snap frozen and processed for Western blotting or immunoflorescence. (A–C) IL-13 signaling in explanted intestinal muscle tissue. (A) Time course for activation of STAT6 by Western blotting or (BC) by immunofluorescence in cancer control tissue. Arrowheads indicate positive nuclei, arrow indicates autoflorescence. (D–F) Double labeling for KIR and PSTAT6 (Panel D), STAT6 (Panel E), or Rα1 (Panel F) in fibrotic CD explanted tissue after 2 h incubation with IL-13. Arrows show KIR^+^ cells which colocalise with IL-13Rα1 but not with PSTAT6 or STAT6. A and C, ×64, B, ×20.

Treatment of explanted muscle tissue with IL-13 induced phosphorylation of STAT6, with maximum activation after 2–4 h (Figure 8A). PSTAT6 was not detected at time 0 (Figure 8B), but was observed in the nucleus of many cells after 2 h (Figure 8C). When fibrotic CD muscle was treated with IL-13, STAT6 was activated in smooth muscle cells, but not in KIR^+^ cells (Figure 8 panel D). Indeed, KIR^+^ cells did not appear to express STAT6 (Figure 8 panel E), despite co-localisation with IL-13Rα1 in explanted muscle (Figure panel F).

### *In vitro* Fibroblast Response to IL-13

**Figure 9:**
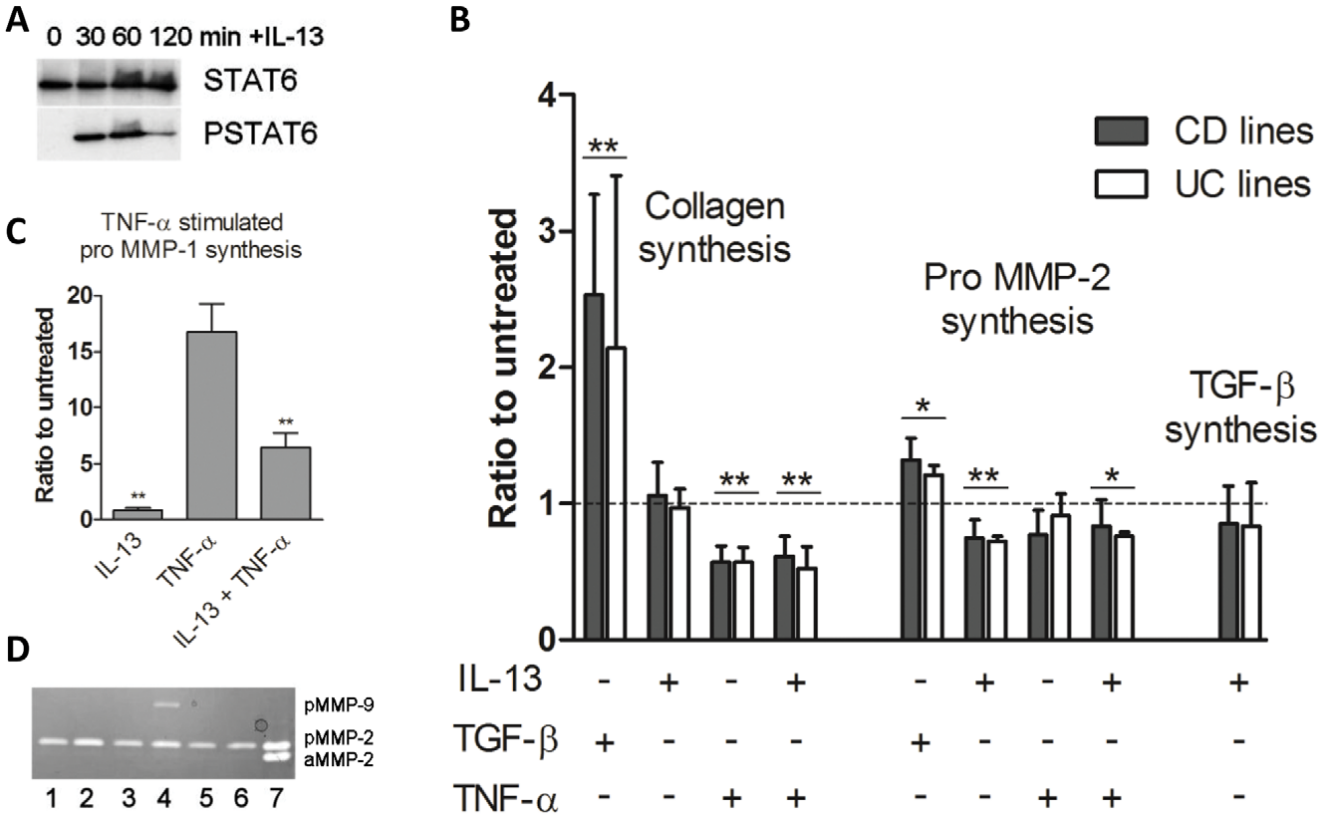
TGF-β drives co-ordinate collagen synthesis and pro-MMP-2 synthesis in intestinal muscle fibroblasts, whereas IL-13 has no effect on collagen synthesis and suppresses proMMP-2, in lines derived from uninvolved CD or UC tissue. (A) Western blots showing phosphorylation of STAT6 by primary fibroblasts in response to exogenous IL-13. Representative blot from 6 experiments. (B) Effect of IL-13, TNF-α and/or TGF-β on collagen, MMP-2 or TGF-β synthesis in primary fibroblasts, Data represent the mean+SEM of the ratio of treated to untreated cultures from a minimum of 8 separate experiments. *p<0.05, **p<0.01 compared to untreated cultures. (C) Effect of IL-13 and/or TNF-α on pro-MMP-1 synthesis. Data represent the mean+SEM of the ratio of treated to untreated cultures from 8 separate experiments. **p<0.01 IL-13+TNF-α compared to TNF-α- or IL-13-stimulated cultures. (D) Zymogram showing the effect of IL-13, TNF-α and/or TGF-β on MMP-2 and MMP-9 synthesis (pMMP = pro MMP, aMMP = active MMP) (lane 1, untreated; lane 2, TGF-β; lane 3, IL-13; lane 4, TNF-α lane 5, TNF-α+IL-13; lane 6, TGF-β+IL-13; lane 7, MMP-2 standard).

Primary muscle-derived mesenchymal cell lines derived from uninvolved regions from both UC and CD, expressed vimentin, type I collagen and prolyl-4-hydroxylase, but less than 1 in 100 cells expressed α-smooth muscle actin (SMA) at passage 3 (Figure S4A–D), and this did not increase with passage number. These cells were, therefore, predominantly fibroblasts and not smooth muscle cells. Cells cultured on microscope slides expressed IL-13Rα1 and IL-13Rα2 (Figure S4E–F). When cultured with IL-13, all primary cell lines phosphorylated STAT6, with maximum activation at 1 h (Figure 9A).

As shown in Figure 9B, there was no significant difference between CD and UC lines in their response to cytokine additions. TGF-β significantly stimulated collagen and proMMP-2 (Figure 9B) synthesis by fibroblasts compared to untreated cultures (p<0.01 for both comparisons). In contrast, IL-13 did not stimulate collagen synthesis in any experiment (p>0.5) and had no significant effect on the amount of total TGF-β secreted into the medium (p = 0.3) (Figure 9B). However, IL-13 significantly suppressed the synthesis of both proMMP-2 (Figure 9B) (p<0.01 compared to untreated) and TNF-α-stimulated proMMP-1 (Figure 9C) (p<0.01 compared to TNF-α stimulation) in all lines. IL-13 also decreased the amount of baseline pro-MMP-1 in unstimulated cultures. Pro-MMP-9 was only detected in TNF-α-stimulated cultures in 6/8 experiments, using the loading conditions appropriate for MMP-2 detection, and was not detected in cultures in the absence of TNFα. In cultures where MMP-9 was detected after TNF-α-stimulation, IL-13 reduced MMP-9 to an undetectable level (Figure 9D).

## Discussion

Intestinal strictures form as a result of chronic inflammation in CD and diverticulitis, but are rarely a clinical problem in UC. Although increased extracellular matrix deposition, including collagen, is driven by inflammation in the mucosa as part of the wound healing response, it is thought that fibrotic strictures in CD result from dysregulated inflammation within the submucosal and muscularis microenvironment, leading to excessive collagen deposition in these tissues [1].

IL-13 has been implicated in both lung and liver fibrosis, though studies examining levels of IL-13 in UC and CD intestine have had contradictory results [27], [28], [29]. No studies have been performed in fibrotic human intestine or in intestinal muscle in IBD. We show here that IL-13 and its receptors are present in the gut, and that they are increased in the outer layers (predominantly muscle) in fibrotic CD intestine compared to other groups, suggesting a role for IL-13 in CD fibrosis. As IL-13 has been proposed as a gut fibrogenic factor [9]–promoting fibrosis by increasing collagen synthesis [4] or TIMP-1 [30], or by decreasing MMP-1 [12]–this is a novel and important finding.

A striking influx or accumulation of IL-13Rα1^++^ KIR^+^ cells, into fibrotic CD muscle tissue was observed. This was a heterogeneous population of cells expressing variable levels of KIR and IL-13Rα1, but the majority of cells expressing these receptors were double positive. The absence of CD3 indicates that these cells are potentially an NK cell subset and not NKT cells. These cells potentially lie on the phenotypic and functional spectrum of type 2 innate lymphoid cell (ILC), a group of lineage-negative lymphoid cells with effector and/or regulatory function, NK characteristics, with plasticity of function, and with cytokine potential biased towards IL-13 and IL-5 [31]. Further studies are underway to fully define their relationship to this lineage and to confirm whether they are contributing to, or responding to, fibrosis. In liver fibrosis, there is evidence for NK cell regulation of fibrosis in early disease [26] through mediation of cytotoxicity against activated collagen-producing stellate cells [32] via IFN-γ, but also enhancement of late-stage disease through secretion of pro-fibrotic factors, including IL-13 [33]. We have consistently observed high level transcription of IL-13 with almost undetectable IFN-γ transcription co-existing in whole fibrotic muscle tissue sections and this coincides with the IL-13^+^, IFN-γ^−^ transcriptional profile of fCD muscle KIR^+^ ILC. Despite cell surface IL-13Rα1 expression, we did not detect IL-13 signalling in stricture KIR^+^ cells, but did find evidence of cell-surface IL-13. Transcription of IL-13 in fibrotic muscle KIR^+^ cells was consistently greater than in adjacent KIR^−^ cells. At present our data point to a role for KIR^+^ cells as a major source of IL-13 and probable net contributors to advanced gut fibrosis in CD.

IL-13 transcription was increased in fibrotic muscle fractions compared to controls. In control tissues, highest levels of collagen was deposited, and therefore synthesised, in submucosa (HVG histology data). In fCD, the submucosa was expanded with a concomitant increase in collagen deposition but there was also a noticeable increase in collagen deposition in the muscle layers. As the amount of muscle-associated submucosa correlated with collagen synthesis but not IL-13 synthesis, and extract IL-13 did not correlate with collagen synthesis, this indicates that muscle is the major source of IL-13 and not the submucosa. As KIR^+^ cells were significantly increased in the muscle layers compared to the submucosa, and IL-13 was expressed by IL-13Rα^+^ KIR^+^ cells, we propose that the increased collagen deposition in the muscle layers is part mediated by IL-13 derived from KIR^+^ cells, acting by downregulation of fibroblast MMP synthesis. The observation that MMP-2 and collagen synthesis correlate in the mucosal fractions, suggest that newly synthesised collagen is remodelled in the mucosa, so preventing a damaging accumulation. A contributory factor to the above scenario may be an increased TIMP-1/MMP-1 ratio in fCD muscle compared to fCD mucosa.

Cultured gut fibroblasts from uninvolved tissue expressed IL-13Rα1 and responded to IL-13 by activation of STAT6. IL-13 did not directly stimulate collagen synthesis, as has been shown for dermal fibroblasts [4]. However, IL-13 may interact differently with fibroblasts derived from fibrotic or inflamed tissue, which were not examined in this study. IL-13 has pro-fibrotic effects via its actions on MMPs, for example inhibiting IL-1β-induced MMP-1 synthesis [12], a key enzyme in initiating degradation of fibrillar collagen. IL-13 significantly reduced both TNF-α-induced MMP-1 and MMP-9, and reduced MMP-2 synthesis by cultured fibroblasts in our study. This is a novel finding, and may indicate the biological consequence of elevated IL-13 in muscle from fibrotic gut, as we observed that MMP-2 synthesis was not co-ordinately up-regulated with collagen synthesis in fibrotic CD muscle. Lack of remodelling does not account for this result, as the correlation was between active collagen synthesis (CICP levels) and MMP-2 levels. Newly synthesised collagen is remodelled by MMP-2, hence the tight coordination of their gene activation, and reduced MMP-2 has been linked to collagen accumulation in liver [34].

The consequence of reduced MMP-1 would be that mature collagen, such as that found in fibrotic lesions, could not be degraded. In extracts, MMP-1 was increased in fibrotic CD muscle but not to the expected level, as the ratio of MMP-1 to collagen synthesis was much higher in iUC and fCD mucosa than in fCD muscle. There was also a significant increase in TIMP-1 in fibrotic muscle tissue, which would have inhibited MMP activity. MMP-9 was decreased in fibroblast cultures exposed to IL-13, but in tissue extracts there was a general increase in all groups compared to cancer controls. MMP-9 is associated with inflammation and leukocyte infiltration, therefore increased leukocyte MMP-9 in diseased tissue extracts may account for this discrepancy.

IL-13Rα1 has been shown to be on epithelial, smooth muscle and nerve cells [35] and to be important in muscle hypercontractility via STAT6 signalling in neurons of the myenteric plexus [7] in mice. In our study, nerve cells and smooth muscle cells (SMC) expressed IL-13Rα1, and SMC signalled in response to exogenous IL-13. IL-13, derived from KIR^+^ cells, may drive muscle hyperplasia in CD by stimulating proliferation of SMC [6], so contributing to stricture formation [5]. We have also shown that nerve cells express IL-13 receptors and are increased in active UC and CD. It is possible that this represents increased innervation of the bowel musculature in IBD, as has been demonstrated for Substance P^+^ nerves [36]. As IL-13 has been shown to act on enteric nerves to stimulate smooth muscle contraction [37], it is likely that increased IL-13 in fibrotic CD muscle promotes both smooth muscle proliferation and contraction, increasing stress within the stricture, further driving fibrogenesis [38] and potentially increasing pain and distress to patients.

In mouse gut, IL-13Rα2 is expressed by mucosal fibroblasts [39] and by cells in the muscle layers [35]. We have shown that Rα2 is expressed at a high level by nerve cells in the muscle, with no obvious expression by other cells in the muscle layers. In fCD muscle, both Rα2 mRNA and protein were significantly increased and may be acting as a either a soluble decoy or stimulatory receptor. We did not find convincing evidence for IL-13Rα1 or Rα2 expression on macrophages in CD muscle, a prerequisite for the IL-13Rα2- profibrotic mechanism proposed by Fichtner-Feigl [9]. However, it is possible that analysis of macrophages by flow cytometry and qPCR, may yield supporting data, as shown in mice [40].

In the muscle layers, our data demonstrate that IL-13 is a potential pro-fibrotic factor in CD, acting by suppressing collagen degradation. We provide evidence of a positive feedback loop, whereby KIR^+^ cells are recruited, by an unknown mechanism, into the muscle layers in CD and then synthesise IL-13, further promoting collagen accumulation and stricture formation. These suggest that drugs targeting the IL-13 pathway should be explored in patients with Crohn’s disease to try to reduce the potential for fibrotic strictures.

## Supporting Information

Figure S1 **Muscle and mucosa fractions after separation.** Representative images of mucosa and muscle fractions from cancer (AB), inflamed UC (CD) and fibrotic CD (EF, E, stitched images). All images captured with x5 objective. Submucosa indicated (sm), dotted line (E) shows muscle/submucosa border in fCD.
(TIF)

Figure S2 **IL-13 receptors are quantitatively increased in strictured muscle.** Image analysis for positive pixel area of (A) total single Rα1^+^, (B) total single Rα2^+^, (C) total Rα1^+^ Rα2^+^ double stained cells. C, Cancer control, uUC, uninvolved UC, iUC, inflamed UC, uCD, uninvolved CD, fCD, fibrotic CD. Data are derived from 14–15 cancer controls, 2 uUC, 8 iUC, 8–12 uCD and 14–21 fCD patients (A, C) or from mucosa (B), * (p<0.05) and ** (p<0.01).
(TIF)

Figure S3 **Transcriptional analysis of fibrotic and non-fibrotic tissues by laser capture microscopy.** A, IL-13 transcripts were assayed in whole frozen tissue sections from fibrotic CD intestine from two patients; B, transcription of IL-13 was compared in KIR^+^ and KIR^−^ cells (500 of each per assay) retrieved by laser capture microscopy from fibrotic CD muscle; C, transcription of IL-13 was compared to transcription of IFN-γ in whole frozen tissue sections from fibrotic CD intestine Representative data from at least three identical assays.
(TIF)

Figure S4 **Primary cell lines are IL-13Rα1^+^ IL-13Rα2^(+)^ fibroblasts.** All cell lines expressed vimentin (AB), prolyl-4-hydroxylase (P4H) (C) and type I collagen (D), with little or no detectable smooth muscle actin (SMA) (A–C). Tissue sections were used as positive controls for SMA stain. Cultured cells also expressed IL-13Rα1 (EG), but IL-13Rα2 was generally expressed at a lower level.
(TIF)

Table S1 **Patients and samples.**
(DOCX)

Table S2 **Primary Antibodies used in immunohistology and Western blotting.**
(DOCX)

Table S3 a) Gene accession numbers and primer sequences. b) Housekeeping genes.
(DOCX)

Table S4 **Mean values for parameters measured in tissue extract data presented in** **Figure 2** **.**
(DOCX)

Table S5 a) Summary of data for proinflammatory and profibrotic parameters in muscle and mucosa from fibrotic Crohn’s disease intestine compared to expression of these parameters in muscle and mucosa in controls. b) Correlation between collagen synthesis and other parameters.
(DOCX)
