# Supplementary figures and images for: IL-13 Promotes Collagen Accumulation in Crohn’s Disease Fibrosis by Down-Regulation of Fibroblast MMP Synthesis: A Role for Innate Lymphoid Cells?
Source: PLoS One. 2012 Dec 31;7(12):e52332. doi: 10.1371/journal.pone.0052332 (PMC3534115; doi:10.1371/journal.pone.0052332)

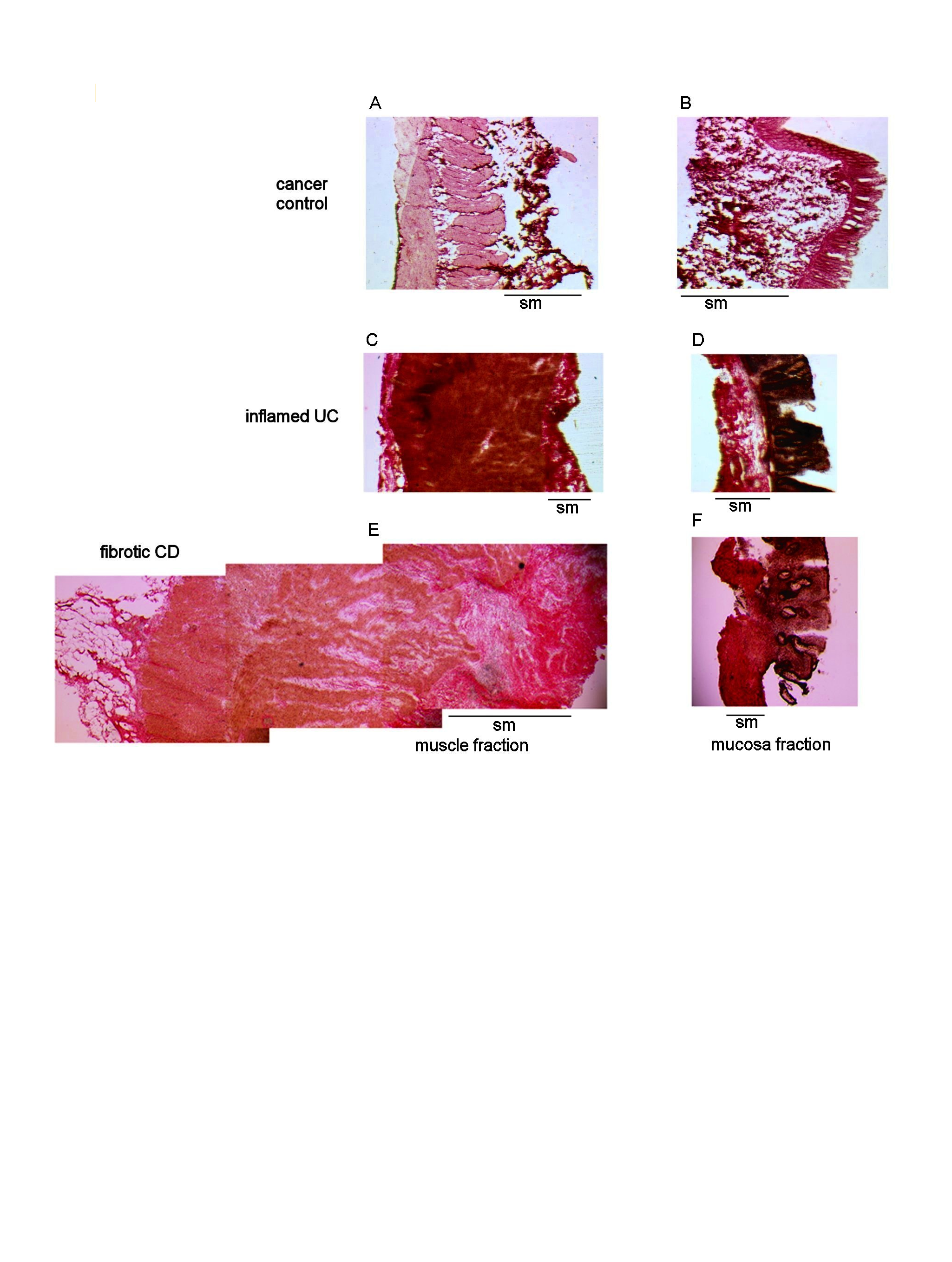

Supplement: Figure S1 — Muscle and mucosa fractions after separation. Representative images of mucosa and muscle fractions from cancer (AB), inflamed UC (CD) and fibrotic CD (EF, E, stitched images). All images captured with x5 objective. Submucosa indicated (sm), dotted line (E) shows muscle/submucosa border in fCD. (TIF) [file pone.0052332.s001.tif]

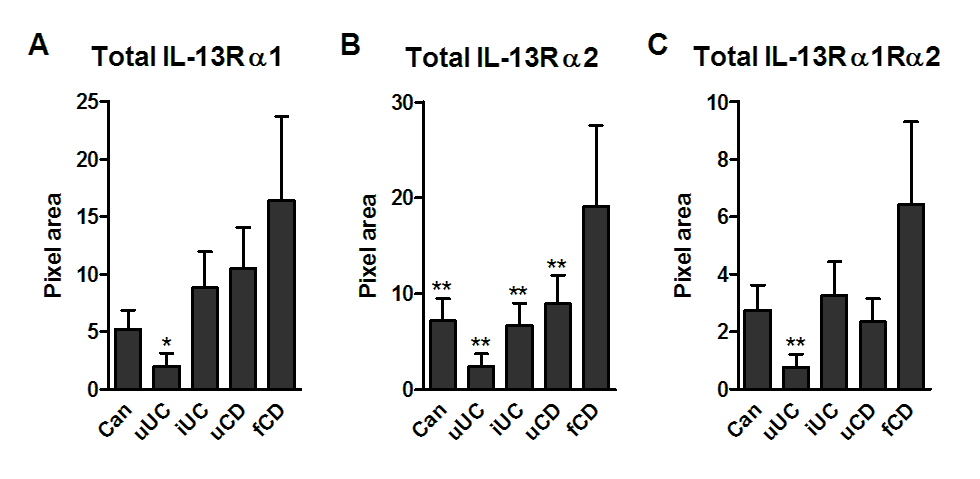

Supplement: Figure S2 — IL-13 receptors are quantitatively increased in strictured muscle. Image analysis for positive pixel area of (A) total single Rα1+, (B) total single Rα2+, (C) total Rα1+ Rα2+ double stained cells. C, Cancer control, uUC, uninvolved UC, iUC, inflamed UC, uCD, uninvolved CD, fCD, fibrotic CD. Data are derived from 14–15 cancer controls, 2 uUC, 8 iUC, 8–12 uCD and 14–21 fCD patients (A, C) or from mucosa (B), * (p<0.05) and ** (p<0.01). (TIF) [file pone.0052332.s002.tif]

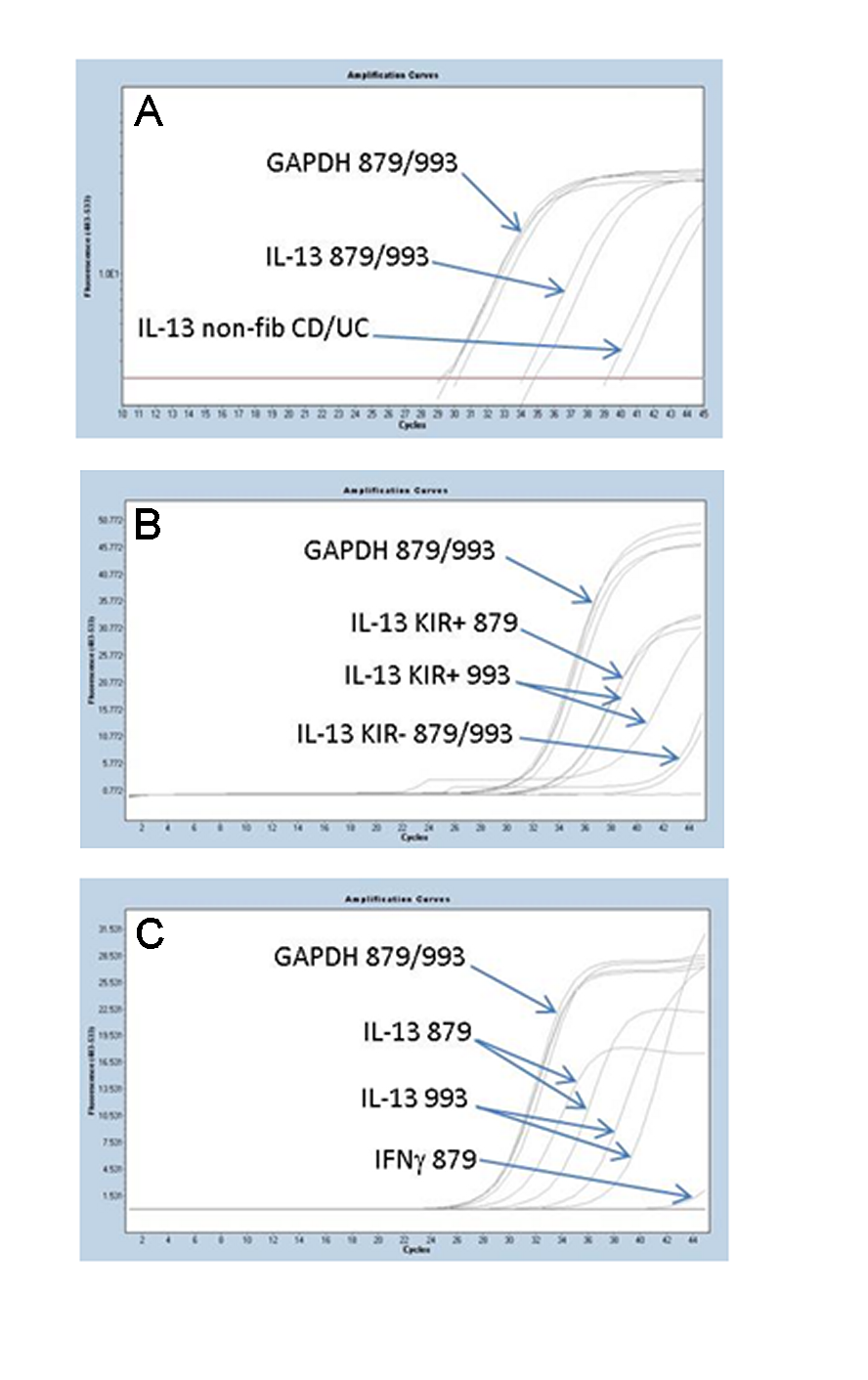

Supplement: Figure S3 — Transcriptional analysis of fibrotic and non-fibrotic tissues by laser capture microscopy. A, IL-13 transcripts were assayed in whole frozen tissue sections from fibrotic CD intestine from two patients; B, transcription of IL-13 was compared in KIR+ and KIR− cells (500 of each per assay) retrieved by laser capture microscopy from fibrotic CD muscle; C, transcription of IL-13 was compared to transcription of IFN-γ in whole frozen tissue sections from fibrotic CD intestine Representative data from at least three identical assays. (TIF) [file pone.0052332.s003.tif]

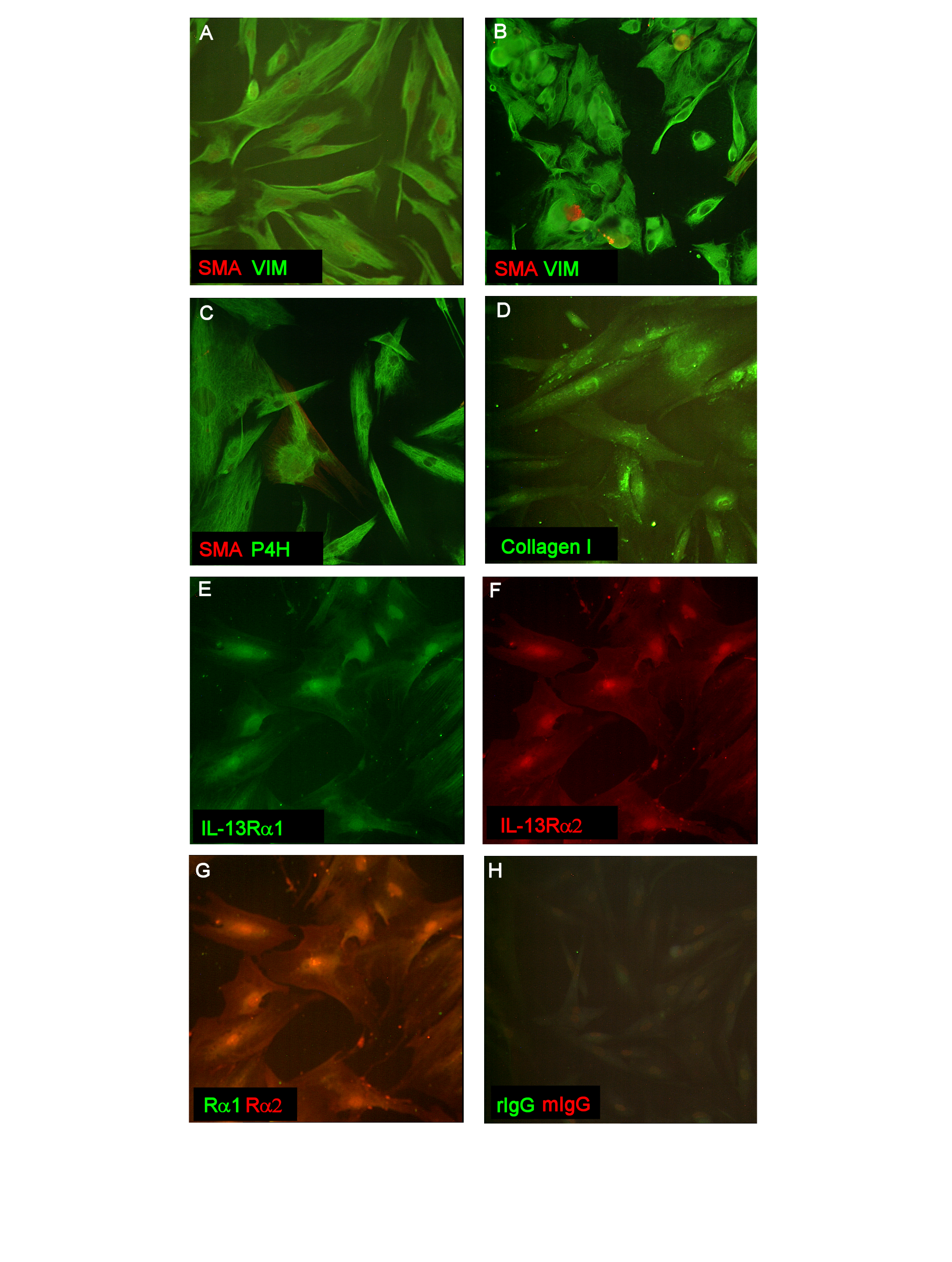

Supplement: Figure S4 — Primary cell lines are IL-13Rα1+ IL-13Rα2(+) fibroblasts. All cell lines expressed vimentin (AB), prolyl-4-hydroxylase (P4H) (C) and type I collagen (D), with little or no detectable smooth muscle actin (SMA) (A–C). Tissue sections were used as positive controls for SMA stain. Cultured cells also expressed IL-13Rα1 (EG), but IL-13Rα2 was generally expressed at a lower level. (TIF) [file pone.0052332.s004.tif]
