# Supplementary material for: IL-13 Promotes Collagen Accumulation in Crohn’s Disease Fibrosis by Down-Regulation of Fibroblast MMP Synthesis: A Role for Innate Lymphoid Cells?
Source: PLoS One. 2012 Dec 31;7(12):e52332. doi: 10.1371/journal.pone.0052332 (PMC3534115; doi:10.1371/journal.pone.0052332)
Supplement: Table S1 — Patients and samples. (DOCX) [file pone.0052332.s005.docx]

Table S1. Patients and samples

| Sample type | Total patient number | Regions and number of samples obtained |
| --- | --- | --- |
| Cancer RM^a^ | 13 | 11 Large intestine |
|  |  | 2 Large intestine + terminal ileum |
| Ulcerative colitis | 8 | 5 inflamed large intestine only  3 Inflamed + uninvolved large intestine |
| Crohn’s disease | 19 | 9 small intestine (3 uCD/fCD, 6fCD only) ^b^ |
|  |  | 6 terminal ileum (4 uCD/fCD, 2 fCD only) |
|  |  | 12 large intestine (2 uCD, 3 uCD/fCD, 7fCD only) |

Total number of patients and the regions of intestine sampled. Multiple samples were obtained from several patients and more than one region was sampled in some cases.

^a^ RM, resection margin.

^b^ The breakdown of samples from Crohn’ patients. uCD, uninvolved CD; fCD, fibrotic CD.
