# Supplementary material for: IL-13 Promotes Collagen Accumulation in Crohn’s Disease Fibrosis by Down-Regulation of Fibroblast MMP Synthesis: A Role for Innate Lymphoid Cells?
Source: PLoS One. 2012 Dec 31;7(12):e52332. doi: 10.1371/journal.pone.0052332 (PMC3534115; doi:10.1371/journal.pone.0052332)
Supplement: Table S2 — Primary Antibodies used in immunohistology and Western blotting. (DOCX) [file pone.0052332.s006.docx]

Table S2. Primary Antibodies used in immunohistology and Western blotting

| Antibody | Isotype | Concentration | Source |
| --- | --- | --- | --- |
| IL-13Rα1 | Rabbit | 1:100 | Santa Cruz |
| IL-13Rα1 | Mouse IgG2b | 1:50 | R&D |
| IL-13Rα2 | Mouse IgG2a | 1:100 | Abcam |
| Prolyl 4-hydroxylase | Mouse IgG1 | 1:40 | Stratech |
| Smooth muscle actin | Mouse IgG2a | 1:100 | Serotec |
| Vimentin | Mouse IgG1 | 1:100 | Serotec |
| CD45 | Mouse IgG2a | 1:100 | AbSerotec |

| Type I collagen | Mouse IgG2b | 1:100 | Southern Biotech |
| --- | --- | --- | --- |
| Macrophages/monocytes | Mouse IgG1 | 1:100 | AbSerotec |
| Mast cell chymase | Mouse IgG1 | 1:250 | AbSerotec |
| Mast cell tryptase | Mouse IgG1 | 1:2000 | AbSerotec |
| CD3 | Mouse IgG1 | 1:100 | R&D |
| CD56 | Mouse IgG2a | 1:100 | AbSerotec |
| KIR | Mouse IgG1 | 1:100 | AbSerotec |
| Neurofilament | Mouse IgG1 | 1:100 | AbSerotec |
| Endothelial cells | Mouse IgG2a | 1:100 | AbSerotec |
| Phospho STAT6 | Rabbit | 1:100 | Cell Signalling |
| STAT6 | Rabbit | 1:100 | Cell Signalling |
| IL-13 | Mouse IgG1 | 1:100 | R&D |
