# Supplementary material for: IL-13 Promotes Collagen Accumulation in Crohn’s Disease Fibrosis by Down-Regulation of Fibroblast MMP Synthesis: A Role for Innate Lymphoid Cells?
Source: PLoS One. 2012 Dec 31;7(12):e52332. doi: 10.1371/journal.pone.0052332 (PMC3534115; doi:10.1371/journal.pone.0052332)
Supplement: Table S3 — a) Gene accession numbers and primer sequences. b) Housekeeping genes. (DOCX) [file pone.0052332.s007.docx]

Table S3a. Gene accession numbers and primer sequences

| Gene | Accession # | Primer sequence |
| --- | --- | --- |
| IL-13 | NM_002188 | Forward: CCTCATGGCGCTTTTGTT  Reverse: CTGGTTCTGGGTGATGTTGA  Probe: (FAM)-TTTGCCTCCCCAGGCCCTGT-(BHQ-1) |
| Il-13Rα2 | NM_000640 | Forward: GGGGAGAGAGGCAATATCAA  Reverse: ATCTCGGTGTCTGAAGATGAAGT  Probe: (FAM)-TCGTTTGCTTGGCTATCGGATGC-(BHQ-1) |
| COL1A | NM_000088 | Forward: CAAGAACCCCAAGGACAAGA  Reverse: CGCCATACTCGAACTGGAA  Probe: (FAM)-TTCGGCGAGAGCATGACCGA-(BHQ-1) |
| TGF-β | NM_000660 | Forward: CTGCCACAGATCCCCTATTC  Reverse: CAGTATCCCACGGAAATAACCT  Probe: (FAM)-CTGGTACCAGATCGCGCCCA-(BHQ-1) |

Table S3b Housekeeping genes

| Housekeeper gene | Abbreviation |
| --- | --- |
| Beta actin | ACTB |
| Beta-2-microglobulin | B2M |
| Glyceraldehyde-3-phosphate dehydrogenase | GAPDH |
| Hydroxymethylbilane synthase | HMBS |
| Hypoxanthine phosphoribosyltransferase 1 | HPRT1 |
| Ribosomal protein L13a | RPL13A |
| Succinate dehydrogenase complex, subunit A | SDHA |
| Tyrosine 3-monooxygenase/tryptophan 5-monooxygenase activation protein, zeta polypeptide | YWHAZ |
