# Supplementary material for: IL-13 Promotes Collagen Accumulation in Crohn’s Disease Fibrosis by Down-Regulation of Fibroblast MMP Synthesis: A Role for Innate Lymphoid Cells?
Source: PLoS One. 2012 Dec 31;7(12):e52332. doi: 10.1371/journal.pone.0052332 (PMC3534115; doi:10.1371/journal.pone.0052332)
Supplement: Table S4 — Mean values for parameters measured in tissue extract data presented in Figure 2 . (DOCX) [file pone.0052332.s008.docx]

Table S4 Mean values for parameters measured in tissue extract data presented in Figure 2

|  |  | cancer | | uUC^3^ | | iUC | | uCD | | fCD | |
| --- | --- | --- | --- | --- | --- | --- | --- | --- | --- | --- | --- |
| **muscle** |  | mean | sem | mean | sem | mean | sem | mean | sem | mean | sem |
| CICP^1^ | ng/ml SP^2^ | 10.96 | 1.62 | 6.24 | 1.72 | 11.04 | 1.76 | 22.95 | 7.60 | 100.74 | 27.11 |
| pMMP-2 | ratio to std | 0.42 | 0.04 | 0.31 | 0.06 | 0.43 | 0.04 | 0.45 | 0.05 | 0.40 | 0.05 |
| pMMP-1 | pg/ml SP | 6.93 | 2.02 | 8.31 | 6.36 | 14.63 | 5.96 | 10.78 | 2.38 | 36.04 | 10.05 |
| TIMP-1 | pg/ml SP | 3165 | 265 | 2900 | 492 | 4088 | 683 | 3374 | 386 | 4797 | 396 |
| pMMP-9 | ratio to std | 2.31 | 0.48 | 4.45 | 1.78 | 3.24 | 0.6 | 3.85 | 0.87 | 3.47 | 0.55 |
| IL-1β | pg/ml SP | 1.4 | 0.76 | 0.03 | 0 | 0.1 | 0.07 | 6.08 | 3.32 | 36.94 | 11.36 |
|  |  | cancer | | uUC | | iUC | | uCD | | fCD | |
| **mucosa** |  | mean | sem | mean | sem | mean | sem | mean | sem | mean | sem |
| CICP | ng/ml SP | 13.4 | 2.45 | 7.69 | 0.3 | 31.37 | 7.59 | 13.95 | 2.38 | 103.3 | 23.86 |
| pMMP-2 | ratio to std | 0.23 | 0.03 | 0.2 | 0.01 | 0.3 | 0.05 | 0.33 | 0.06 | 0.63 | 0.11 |
| pMMP-1 | pg/ml SP | 7.3 | 1.56 | 8.77 | 2.73 | 162.63 | 68.62 | 15.63 | 4.31 | 84.68 | 27.76 |
| TIMP-1 | pg/ml SP | 3221 | 236 | 3969 | 1130 | 6904 | 1426 | 3018 | 268 | 4893 | 504 |
| pMMP-9 | ratio to std | 1.39 | 0.3 | 2.6 | 0.61 | 3.31 | 0.47 | 2.24 | 0.39 | 2.45 | 0.47 |
| IL-1β | pg/ml SP | 6.09 | 3 | 1.68 | 0.97 | 33.02 | 10.78 | 11.5 | 3.72 | 63.72 | 14.65 |

^1^ Abbreviations: CICP, Type I collagen carboxy-terminus peptide as a measure of collagen synthesis; MMP, matrix metalloproteinase; TIMP, tissue inhibitor of metalloproteinases; IL-1β, interleukin -1β.

^2^ SP, concentration of analyte per ml standardised soluble protein

^3^ uUC, uninvolved UC; iUC, inflamed UC; uCD, uninvolved CD; fCD, fibrotic CD
