# Supplementary material for: IL-13 Promotes Collagen Accumulation in Crohn’s Disease Fibrosis by Down-Regulation of Fibroblast MMP Synthesis: A Role for Innate Lymphoid Cells?
Source: PLoS One. 2012 Dec 31;7(12):e52332. doi: 10.1371/journal.pone.0052332 (PMC3534115; doi:10.1371/journal.pone.0052332)
Supplement: Table S5 — a) Summary of data for proinflammatory and profibrotic parameters in muscle and mucosa from fibrotic Crohn’s disease intestine compared to expression of these parameters in muscle and mucosa in controls. b) Correlation between collagen synthesis and other parameters. (DOCX) [file pone.0052332.s009.docx]

Table S5a. Summary of data for proinflammatory and profibrotic parameters in muscle and mucosa from fibrotic Crohn’s disease intestine compared to expression of these parameters in muscle and mucosa in controls

|  | Comparison to uninvolved CD | | | | Comparison to cancer margins | |
| --- | --- | --- | --- | --- | --- | --- |
| Parameter | Mucosa | Muscle |  | Mucosa | | Muscle |
| Collagen synthesis | ↑** | ↑** | ↑** | | | ↑** |
| MMP-2 (pro) | ↑** | 0.67 | ↑** | | | 0.68 |
| MMP-9 (pro) | 0.64 | 0.33 | ↑0.13 | | | 0.32 |
| MMP-1 (pro) | 0.66 | 0.54 | 0.64 | | | 0.33 |
| TIMP-1 | ↑* | ↑** | ↑0.08 | | | ↑** |
| IL-1β | ↑0.12 | ↑** | ↑** | | | ↑** |

Normal tissue was taken from colorectal cancer resection, or from Crohn’s patients without inflammatory infiltrate or signs of fibrosis.^a^

^a^ Includes tissues from both small bowel and large bowel resection – details in text

^b^ Direction arrows indicate direction of significant change or trend from relevant control

^c^ Asterisks denote level of significant change from relevant control: * p<0.05; ** p<0.01

Table S5b. Correlation between collagen synthesis and other parameters

| Parameter | Mucosa | Muscle |
| --- | --- | --- |
| Pro-MMP2 | 0.003 | 0.787 |
| IL-13 mRNA | 0.138 | 0.329 |
| TIMP-1 | 0.074 | 0.0001 |
| Pro-MMP-1 | 0.011 | 0.052 |
| Pro-MMP-9 | 0.445 | 0.478 |
| IL-1β | 0.001 | 0.0001 |

Significance of correlations between type I collagen synthesis and inflammation and fibrosis-related parameters in separated muscle and mucosal fragments from all tissue groups. Pearson correlation coefficients determined pair-wise by patient for each tissue type
